# Disentangling sex-specific mechanisms in neuropathic and nociplastic chronic pain: a review

**DOI:** 10.3389/fneur.2025.1730291

**Published:** 2026-01-09

**Authors:** Sven Vanneste, Jorge Castejón-España, Elva Arulchelvan, Dirk De Ridder

**Affiliations:** 1 School of Psychology, Trinity College Dublin, Dublin, Ireland; 2 Global Brain Health Institute, Trinity College Dublin, Dublin, Ireland; 3 Trinity College Institute of Neuroscience, Trinity College Dublin, Dublin, Ireland; 4 Section of Neurosurgery, Department of Surgical Sciences, Dunedin School of Medicine, University of Otago, Dunedin, New Zealand

**Keywords:** chronic pain, neuropathic pain, nociplastic pain, precision pain therapeutics, sex differences

## Abstract

Chronic pain is a multidimensional condition shaped by sex-specific biological and sociocultural factors, leading to distinct vulnerabilities, mechanisms, and treatment experiences in men and women. While women consistently exhibit lower pain thresholds, more unpleasantness, and higher prevalence of chronic pain syndromes, these differences extend beyond sensory experience and reflect qualitative divergences in immune signalling, hormonal modulation, brain network engagement, and psychosocial processing. Emerging preclinical and clinical evidence demonstrates that neuropathic pain in males is predominantly driven by microglia-dependent neuroinflammation, whereas in females it is sustained by adaptive immune mechanisms involving T-cell signalling. In nociplastic pain syndromes—such as fibromyalgia—women-biased hormonal fluctuations, limbic hyperconnectivity, and stress–immune interactions amplify central sensitization and affective suffering. Genetic studies further reveal largely non-overlapping sex-specific risk loci and gene expression patterns in pain-related tissues, supporting divergent molecular trajectories toward chronic pain. Despite these mechanistic differences, current treatments largely target sex-indifferent nociceptive circuits, resulting in comparable analgesic outcomes but sex-specific side-effect profiles and device tolerability. This review synthesizes converging evidence across genetic, neural, immune, hormonal, psychosocial, and clinical domains to propose a dual-framework model: chronic pain emerges from shared core pathways but is differentially modulated by sex-specific upstream mechanisms. Recognizing these distinctions opens a path toward hybrid treatment strategies that combine universal interventions with sex-tailored adjuncts, offering a foundation for precision pain therapeutics.

## 1 Introduction

Pain is commonly defined as an unpleasant sensory and emotional experience associated with, or resembling that associated with, actual or potential tissue damage (1, 2). Pain is the most common reason patients seek medical care, and chronic pain represents a significant burden on healthcare systems worldwide (3). Chronic pain refers to pain that persists beyond the normal healing time of the original injury and therefore loses the protective role of acute nociception (4). It is not merely a prolonged form of acute pain but is driven by distinct underlying mechanisms (5). Whereas acute pain is usually considered a symptom of another condition, chronic pain—defined by the IASP and ICD-11 as pain lasting more than three months regardless of cause (4, 6).

Historically, research has assumed a largely “sex-neutral” model of pain. Yet mounting evidence demonstrates that men and women differ substantially in pain perception, processing, and response to treatment (7–9). These differences span the entire biopsychosocial spectrum, including neural circuitry, immune function, hormonal regulation, genetics, and social context (10). Understanding these distinctions is critical, as failure to account for sex as a biological variable risks overlooking mechanisms unique to half of the population and may lead to suboptimal or even harmful therapeutic approaches. In this review, we aim to disentangle the current understanding of sex-specific mechanisms in pain, tracing their influence from the acute to the chronic pain state. Table 1 summarizes key sex differences in acute, neuropathic, and nociplastic chronic pain.

**Table 1:** Key considerations for sex differences in acute, neuropathic, and nociplastic chronic pain.

Domain	Women (relative to men)	Men (relative to women)	Key considerations for research & clinical care
*Prevalence and clinical burden*	Higher prevalence of most chronic pain syndromes; lower pain thresholds and tolerance; greater pain unpleasantness and broader impact on quality of life; higher rates of comorbid anxiety and depression.	Lower prevalence and generally higher pain thresholds; pain may be underreported due to stoicism and gender norms.	Epidemiologic and clinical studies should routinely stratify by sex; screening for comorbid affective disorders is particularly critical in women, while under-recognition of pain may be an issue in men.
*Transition from acute to chronic pain*	Greater vulnerability to pain chronification, especially after puberty; stronger influence of early-life adversity and pain catastrophizing; more widespread and persistent hyperalgesia in several preclinical models.	Lower overall risk of chronification but still vulnerable under specific biological and psychosocial contexts.	Longitudinal studies should examine sex-specific predictors (hormonal stage, early-life stress, catastrophizing); prevention strategies may need different emphasis in women vs. men.
*Neural circuitry and brain networks*	More consistent alterations in insula and primary sensorimotor regions; stronger coupling of structural/functional changes with emotional and catastrophizing dimensions of pain; greater limbic and medial pain pathway engagement in nociplastic conditions.	In some chronic pain states, greater disruption of reward circuitry and reduced ventral striatal activation; structural changes more linked to beliefs about control and fear of movement.	Neuroimaging should move beyond “average” effects to map sex-specific network engagement and its cognitive–emotional correlates; may inform sex-tailored targets for neuromodulation and psychotherapy.
*Immune mechanisms*	Chronic pain more strongly linked to adaptive immune responses (e.g., CD4^+^ T-cell–driven mechanisms, IL-17A, IFN-γ) and broader immune–affective crosstalk.	Neuropathic and inflammatory pain more strongly driven by spinal microglial activation (e.g., P2X4/P2X7 signalling, TNF-α, IL-1β, BDNF).	Preclinical and translational work should explicitly test microglia- vs. T-cell–targeted interventions by sex; immune signatures may also inform selection of anti-inflammatory or immunomodulatory adjuncts.
*Hormonal modulation across the lifespan*	Ovarian hormones (oestrogens, progesterone) dynamically modulate pain sensitivity and central sensitization; pubertal onset, menstrual cycle, pregnancy, and menopause are key inflection points for pain risk and experience.	Testosterone exerts broadly antinociceptive and anxiolytic effects and may protect against chronic widespread pain; andropause is understudied in relation to pain.	Clinical and preclinical studies should incorporate hormonal status (cycle phase, contraceptives, pregnancy, menopause/andropause) and consider hormone-based or hormone-informed analgesic strategies.
*Sociocultural and psychosocial factors*	Fewer social barriers to reporting pain but higher risk of dismissal or psychologization in clinical encounters; greater catastrophizing and higher exposure to trauma and caregiving-related stress; normalization of reproductive pain.	Stronger norms favouring stoicism and underreporting; help-seeking may be delayed, and pain framed more in functional or performance terms.	Pain assessment must account for gendered expression and clinician bias; interventions should target catastrophizing and stress differently across sexes and address structural inequities in access to care.
*Neuropathic vs nociplastic mechanisms*	Neuropathic: greater contribution of T-cell–mediated mechanisms; Nociplastic: higher prevalence (e.g., fibromyalgia), stronger limbic hyperconnectivity, stress–immune interactions, and hormone-sensitive central sensitization.	Neuropathic: predominance of microglial-driven neuroinflammation; Nociplastic: may show relatively greater sensory-discriminative than affective weighting.	Same clinical syndrome may arise via distinct biological routes in men vs. women. Mechanism-based phenotyping should incorporate sex when selecting immunological, hormonal, or CNS-targeted treatments.
*Treatment efficacy, side effects and device tolerability*	Overall similar analgesic efficacy to men for standard pharmacological, psychotherapeutic, surgical, and neuromodulation interventions, but higher rates of adverse drug reactions and some neuromodulation explants or revisions.	Comparable analgesic benefit but different perioperative risk profiles (e.g., cardiovascular and infectious complications) and possibly different expectations around device use and outcomes.	Current “sex-neutral” treatments likely target shared nociceptive circuits; adding sex-specific adjuncts (e.g., microglia vs. T-cell modulators, hormone-informed strategies, tailored counselling) may enhance benefit–risk balance.
*Implications for precision pain therapeutics*	Pain often shaped by convergence of limbic, hormonal, immune, and sociocultural influences; may benefit particularly from therapies that combine central, immune, and psychosocial targets.	Pain more often framed through sensory, functional, and reward-related disruptions; may benefit from strategies that emphasize motivational, behavioural, and microglial targets.	A dual-framework model—core shared pathways plus sex-specific upstream modulators—supports hybrid regimens: universal interventions (e.g., SNRIs, CBT, neuromodulation) combined with sex-tailored adjuncts based on mechanistic profiles.

## 2 Sex-specificity in nociceptive pain

A consistent body of evidence indicates that women report lower pain thresholds and tolerance compared with men, and tend to rate pain as more intense and unpleasant (9, 11–13). Women also exhibit greater temporal summation of pain, while men generally show stronger conditioned pain modulation (14). Physiological evidence further corroborates these behavioural and self-report data. Women display stronger autonomic nervous system reactivity to pain, including increased galvanic skin conductance and enhanced pupillary dilation, reflecting heightened sympathetic activation (15). These autonomic differences are not restricted to experimental conditions but are also observed in clinical contexts, such as chronic low back pain (16). In addition, women are more likely to engage in pain catastrophizing, which amplifies both sensory and affective dimensions of the pain experience (13). However, the causal pathways remain uncertain: it is not yet clear whether elevated sympathetic responses exacerbate emotional distress, or whether greater emotional reactivity drives the observed autonomic changes. Although the evidence points to sex differences, where women generally exhibit greater pain sensitivity than men, variability in effect sizes across methodologies and outcomes has contributed to differing interpretations in the literature.

## 3 Sex-specificity in chronic pain

The divergence between sexes is even more striking in chronic pain (17). Women are disproportionately affected by nearly all major chronic pain syndromes, including migraine, fibromyalgia, rheumatoid arthritis, musculoskeletal pain, complex regional pain syndrome (CRPS), temporomandibular disorders, and postoperative pain (7, 9, 18).

Beyond prevalence, women report higher pain intensity and lower quality of life within the same diagnostic categories (18, 19). They also utilize healthcare services more frequently for both painful and non-painful disorders, suggesting broader suffering (9). These differences extend into mental health: women have approximately twice the lifetime prevalence of depression and most anxiety disorders, with subclinical symptoms being more common as well.

The emotional consequences of chronic pain diverge by sex. Women’s higher pain intensity is more closely linked to frustration and fear, whereas in men it is associated with anxiety and depression (20). Similarly, women’s perception of greater pain unpleasantness leads to depression and frustration, while men primarily report frustration (20). This aligns with broader patterns of stress reactivity, where women tend to perceive stressful life events as more intense (21), contributing to their elevated rates of anxiety, depression, and post-traumatic stress disorder (PTSD) in contexts such as cancer (22), trauma (92), and the COVID-19 pandemic (23).

Despite these robust clinical differences, preclinical pain research has historically relied overwhelmingly on male animals (24). For example, 80% of studies published in *PAIN* in 2015 used only male rodents (25). Clinical studies often fail to analyse sex as a biological variable, perpetuating a knowledge gap that undermines our ability to develop effective treatments for women (26). However, since the implementation of sex-inclusive research policies by funding agencies in 2014–2016, there has been a gradual increase in the inclusion of both sexes and in sex-disaggregated analyses, although substantial gaps remain in fully integrating sex as a biological variable in pain research (27, 28).

## 4 Biological basis of sex differences in chronic pain

### 4.1 Differences in the transition from acute to chronic pain

Research has identified factors contributing to the progression from acute to chronic pain. However, the potential for sex-based differences in these factors remains insufficiently understood. While more than 90% of clinical studies include both men and women, the majority fail to conduct sex-stratified analyses (25, 29). Evidence from adolescent populations indicates that female sex is a consistent predictor for the development of chronic pain (30), a pattern not observed in younger children (31). This is consistent with the possibility that sex hormones introduced during puberty may influence the trajectory toward chronic pain (29). Supporting this, women have been shown to exhibit higher rates of chronic pain following acute pain episodes in emergency departments and after thoracic surgery compared with men (32, 33). Additionally, early life exposure to trauma or stress may predispose women more strongly than men to visceral pain syndromes such as irritable bowel syndrome (34).

Psychosocial predictors of pain chronification have received less attention in the context of sex differences. One research paper indicates that women report higher levels of pain catastrophizing, characterized by magnification, rumination, and helplessness, compared with men (32). That is, catastrophizing predicted chronic pain development in both sexes when pain intensity exceeded a minimal threshold, but predicted persistence of pain only in women when higher intensity thresholds were applied (32). Moreover the same study reveals that pre-existing chronic pain predicted poorer postoperative outcomes in women but not in men (32). Whether other psychosocial predictors exhibit sex-specific patterns remains largely unexplored.

To further understand the underlying biological mechanisms in sex differences in the transition from acute to chronic pain, it is necessary to also draw on insights from animal research. One study using a preclinical animal model that relies on a two-stage process involving a priming stimulus (e.g., inflammation, surgery, stress, opioid exposure, or early-life insult) followed by an induction stimulus (e.g., subsequent injury or stressor) showed that while induction stimuli often evoke minimal responses in naïve animals, primed animals elicit prolonged and exaggerated pain responses, thereby modelling chronic pain (17). Sex differences have begun to emerge in these paradigms. For instance, inflammatory priming (with carrageenan or complete Freund’s adjuvant) produced hyperalgesia in men but not women following subsequent induction (35). Conversely, priming with proinflammatory cytokine interleukin 6 (IL-6) or paw incision produced equivalent hyperalgesia responses across sexes (36). Non-inflammatory priming, such as repeated acidic saline injections, induced chronic hyperalgesia in both sexes, though the severity and distribution of hyperalgesia were greater in women (37). Models of ischemia–reperfusion injury and activity-induced hyperalgesia similarly demonstrated prolonged and more widespread pain behaviours in women compared to men (17).

Stress-related priming also highlights sex-specific vulnerability. Repeated stress combined with nitric oxide donor (a compound that releases nitric oxider, a molecule involved in many physiological processes like blood pressure control and immune response) induced migraine-like symptoms in both sexes, whereas stress alone induced muscle and visceral hypersensitivity only in males in some animal models (38, 39). Early-life stress paradigms, including neonatal inflammation, chemotherapy exposure, or procedural pain, often produced exaggerated pain responses upon re-injury in adulthood, with notable sex differences (40). These findings align with human studies suggesting that adverse early-life experiences confer greater long-term pain risk in women (41).

Alterations in endogenous inhibitory systems further contribute to sex-specific pain chronification. In latent sensitization models, hyperalgesia can be reinstated by blocking opioid receptors after resolution of the initial insult. Interestingly, spinal *κ*-opioid receptor blockade produced more robust reinstatement of hyperalgesia in women compared with men, whereas *μ*- and *δ*-opioid receptor inhibition affected both sexes equally (37).

In conclusion, the transition from acute to chronic pain is shaped by a complex interplay of biological, psychosocial, and environmental factors, with accumulating evidence pointing toward meaningful sex differences across these domains. While women appear more vulnerable to pain chronification in both clinical and preclinical contexts, the underlying mechanisms are not fully explored. Sex hormones, early-life stress, and differential engagement of endogenous inhibitory systems may all contribute to this disparity, alongside psychosocial influences such as pain catastrophizing.

### 4.2 Structural and functional brain differences

Structural and functional brain differences between men and women modestly modulate vulnerability to, and the experience of, chronic pain, against a background of substantial overlap and strong hormonal and psychosocial influences (13, 42). Chronic pain is associated with long-term changes across distributed brain network, including sensory-discriminative (primary and secondary somatosensory cortex, posterior insula), affective–motivational (anterior insula, anterior cingulate cortex, amygdala), cognitive-control (prefrontal and parietal cortices), descending modulatory (periaqueductal gray and related brainstem nuclei), and reward-related regions (ventral striatum, ventral tegmental area) (13, 43).

Structurally, chronic pain in both sexes is associated with reduced gray matter volume and cortical thickness in prefrontal, cingulate, insular, thalamic, and primary somatosensory regions, and with altered white-matter connectivity (13, 44, 45). Several studies indicate that, in women, abnormalities in primary sensorimotor cortex and insula are more prominent or consistent (46, 47). In chronic spinal pain, gray matter morphology in sensorimotor, parietal, orbitofrontal, and insular regions shows different psychological correlates by sex: in women, larger regional volumes relate more strongly to perceived chronicity and consequences of pain, emotional illness representations, and catastrophizing, whereas in men, similar regions are more tightly linked to beliefs about personal control and fear of movement (48). Thus, sex differences may lie less in which regions are affected than in how structural changes couple to cognitive–emotional aspects of pain (45).

Chronic pain also interacts with reward and descending modulatory circuitry in a sex-dependent manner (49). In reward-anticipation tasks, healthy men often show stronger ventral striatal activation than women, but in chronic back pain this response is selectively blunted in men, potentially contributing to reduced motivation (50). Women, who are generally more pain-sensitive, sometimes show reduced engagement of the periaqueductal gray and prefrontal–brainstem inhibitory pathways during active pain-inhibition tasks, whereas men may recruit these circuits more robustly (50, 51).

Neurochemically, transmitter systems involved in chronic pain and affective disturbance are regulated in a sex-dependent manner by gonadal hormones, and sexually dimorphic development of cortical–limbic and brainstem circuits may contribute to higher rates of internalizing symptoms in women, different externalizing patterns in some men, and sex-specific treatment responses (36, 52).

### 4.3 Sex-specific mechanisms in neuropathic vs. nociplastic chronic pain

Neuropathic and nociplastic pain represent two of the most clinically challenging chronic pain categories, and both reveal profound sex differences in underlying biology (53). Although they share features such as central sensitization, hyperexcitability of pain pathways, and maladaptive neuroimmune signalling, the mechanisms that drive these states differ markedly between men and women, suggesting that the same clinical presentation can emerge through divergent biological routes (25, 52, 53).

Neuropathic pain, which develops after direct injury to the peripheral or central nervous system, such as in diabetic neuropathy, chemotherapy-induced neuropathy, or postherpetic neuralgia, has traditionally been studied in male animal models (24, 25). Findings from this research consistently demonstrate that microglial activation in the spinal cord plays a dominant role in maintaining hypersensitivity in males (54). Activated microglia release proinflammatory cytokines and neuromodulators that amplify nociceptive transmission and promote chronic pain (55). In contrast, parallel studies in female animals indicate that adaptive immune cells, particularly T lymphocytes, assume a more central role in driving hypersensitivity (56). This divergence suggests that men and women may develop neuropathic pain through distinct immune pathways, which has direct therapeutic implications. Treatments that suppress microglial activity may be particularly beneficial for men, whereas interventions that modulate T-cell activity or peripheral immune signalling could prove more effective for women (57). Importantly, these mechanistic differences highlight why certain preclinical drug candidates that appeared promising in male models failed to translate effectively in women.

Nociplastic pain, in contrast, does not originate from obvious nerve damage or tissue injury but arises from maladaptive changes in central nervous system processing (58). Conditions such as fibromyalgia and chronic widespread pain are prototypical examples, and they are disproportionately diagnosed in women, in some cases with female-to-male prevalence ratios exceeding 9:1 (59). Mechanistic studies suggest that several factors converge to produce this imbalance. Fluctuations in ovarian hormones, particularly oestrogen, appear to sensitize excitatory neurotransmission and reduce the efficacy of descending inhibitory circuits, thereby amplifying the central nervous system’s response to otherwise non-noxious stimuli (60, 61). Neuroimaging studies in women with nociplastic pain syndromes reveal enhanced connectivity between limbic-emotional regions such as the anterior cingulate cortex and insula and the medial pain pathway, which heightens the affective dimension of pain (13). Men with comparable disorders often demonstrate more engagement of sensory-discriminative regions, producing a different qualitative pain experience. Stress physiology also plays a role: women exhibit stronger hypothalamic–pituitary–adrenal axis reactivity, which may contribute to sustained immune dysregulation and perpetuate nociplastic pain through chronic low-grade inflammation and abnormal cortisol signalling (62).

Neuropathic pain is often maintained by sex-specific immune pathways, while nociplastic pain is shaped more strongly by hormonal modulation, altered brain network connectivity, and stress–immune interactions. In both cases, the end result is persistent and debilitating pain, but the underlying biology is not the same for men and women. Recognizing these distinctions is critical for advancing precision medicine. Pharmacological approaches that target microglial signalling may succeed in male neuropathic pain patients but offer little relief in women, who may instead benefit from therapies directed at T-cell activity or hormonal modulation. Similarly, treatments that are effective for nociplastic pain in women—such as serotonin-norepinephrine reuptake inhibitors, exercise-based interventions, and cognitive-behavioural therapy—may not translate directly to men, whose pain is less tied to limbic hyperconnectivity and hormonal fluctuations. Disentangling these sex-specific mechanisms is not only a matter of biological insight but also a clinical necessity, as it promises to reduce the trial-and-error approach that characterizes much of current pain management and to deliver therapies that are more closely matched to the underlying drivers of pain in each sex.

### 4.4 Genetic and epigenetic influences

Sex differences in brain anatomy and function are partly genetically encoded. Roughly 37% of genes show sex-biased expression in at least one tissue, with the brain and spinal cord among the most differentiated (63). Building on this, one of the largest sex-stratified genome-wide association studies (GWAS) of multisite chronic pain to date, analysed data from 178,556 men and 209,093 women in the UK Biobank (64). Their results revealed striking sex-specific genetic architectures. In men, 123 single nucleotide polymorphisms (SNPs) across five independent loci were significantly associated with multisite chronic pain, whereas in women 286 SNPs across ten loci reached genome-wide significance. Only one gene, DCC, overlapped between sexes, highlighting largely distinct sets of risk genes. A meta-analysis of sex-stratified results identified an additional 87 significant SNPs, including 11 novel loci not previously linked to chronic pain (64). Multisite chronic pain was moderately heritable, with SNP-based heritability estimated at ~12.5% in women and ~10.6% in men. Although the genetic correlation between men and women chronic pain was high (rg = 0.92), it was significantly less than unity, indicating overlapping yet partially distinct genetic risk factors.

Gene expression analyses showed that nearly all multisite chronic pain-associated genes were expressed in the dorsal root ganglion, underscoring its central role in nociceptive processing (64). Women-specific risk genes were enriched for expression in brain regions such as the cerebellum and frontal cortex, while no significant tissue enrichment was detected for men-specific genes—possibly due to differences in effect size or statistical power. The study also revealed sex-specific pleiotropy: multisite chronic pain in men and women exhibited distinct genetic correlations with psychiatric, autoimmune, and anthropometric traits.

Importantly, sex-specific polygenic risk scores predicted chronic widespread pain in both sexes, but with differing magnitudes and association patterns, suggesting sex-differential pathways connecting localized and widespread pain (64). Together, these findings highlight contributions from both central nervous system and immune system processes, while reinforcing that sex shapes genetic vulnerability at multiple levels—SNPs, genes, and transcript abundance.

### 4.5 Immune mechanisms

The immune system contributes to pain processing in a sex-dependent manner (65). In male rodents, spinal microglia are the primary mediators of hypersensitivity in neuropathic and inflammatory pain. Microglia become activated following nerve injury, upregulating purinergic P2X4 and P2X7 receptors, and releasing pro-inflammatory cytokines such as TNF-*α*, IL-1β, and BDNF, which enhance excitatory synaptic transmission in dorsal horn neurons (66–69). In contrast, female rodents rely less on microglial signalling and more on adaptive immune mechanisms, particularly CD4^+^ T- cells (56). These T-cells infiltrate the spinal cord and meninges, releasing IL-17A, interferon-*γ*, and other mediators that sustain central sensitization (66, 67).

Interestingly, in bone cancer pain, microglial activation appears to play a critical role in both sexes, but the contribution of T-cells shows a male bias, suggesting that immune–neural crosstalk may shift depending on disease context (70). These sex-dependent immune signatures extend further beyond pain into affective disorders. Transcriptomic studies reveal sex-specific patterns of microglial gene regulation, with men showing stronger pro-inflammatory transcriptional responses, while women exhibit more adaptive immune-linked expression profiles (13, 71). Such immune differences may influence the chronicity and emotional salience of pain, as persistent neuroinflammation can alter monoaminergic and neurotrophic signalling in brain regions implicated in mood regulation. In turn, shared inflammatory mediators such as IL-1β and TNF-*α* may simultaneously drive nociceptive sensitization and depressive-like behaviours, highlighting immune dysregulation as a common biological substrate (72). Given the high comorbidity between chronic pain and depression, these findings suggest that immune mechanisms may provide a mechanistic bridge between nociceptive processing and affective suffering.

### 4.6 Hormonal modulation of pain

Sex hormones exert critical and dynamic influences on nociception and pain modulation across the lifespan. Prior to puberty, boys and girls display comparable thresholds for pain detection and similar ratings of unpleasantness. With the onset of puberty, however, sexual dimorphism in pain emerges: girls develop lower pain thresholds and exhibit a higher prevalence of recurrent and chronic pain conditions (9, 14). In adulthood, women consistently demonstrate greater experimental pain sensitivity and higher rates of clinical pain syndromes relative to men (24, 25). After menopause, these sex differences diminish, implicating ovarian hormones as key mediators of women-biased pain vulnerability (73).

Many researchers describe testosterone as generally helping to reduce how strongly people experience pain (73). Higher endogenous testosterone levels are associated with reduced postoperative analgesic requirements, decreased prevalence of chronic widespread pain, and improved mental health outcomes (17). Experimental testosterone replacement in hypogonadal men attenuates pain intensity, unpleasantness, fear, and anxiety during noxious stimulation, effects that appear to involve facilitation of descending inhibitory control and suppression of central sensitization (74). This suggests that testosterone exerts both antinociceptive and anxiolytic effects, modulating sensory-discriminative as well as affective-motivational dimensions of pain.

Progesterone, by contrast, appears to exert subtler and more dissociative effects. Evidence indicates that progesterone may decouple pain intensity from its affective unpleasantness, suggesting a selective influence on neural substrates underlying the emotional appraisal of nociceptive input (75). Oestrogens add further complexity, as their actions are dose-dependent and context-dependent: at low concentrations, oestradiol facilitates nociception via excitatory glutamatergic signalling and neuroinflammatory cascades, whereas at higher concentrations, it engages opioid and serotonergic pathways to produce antinociceptive effects (76).

Taken together, these findings highlight that sex differences in pain perception are not static but dynamically regulated by fluctuating hormonal milieus. The differential actions of testosterone, progesterone, and oestrogens underscore the need to conceptualize pain as a hormonally sensitive phenomenon shaped by both peripheral and central mechanisms. Elucidating these processes has significant translational implications, providing a mechanistic basis for sex-specific vulnerability to chronic pain disorders, informing individualized analgesic strategies, and opening the possibility of hormone-based interventions for pain management.

## 5 Sex differences in treatment outcomes for chronic pain

Despite the significant biological and psychosocial differences in pain mechanisms between men and women, most current therapeutic modalities—including pharmacological interventions, psychotherapy, surgery, and neuromodulation—show no substantial sex-related differences in efficacy when evaluated through standard outcome measures (53). However, meaningful sex-based differences do emerge in the profile of adverse effects and complications associated with these treatments (77).

### 5.1 Pharmacological treatments

Analgesic medications, including opioids and non-opioid agents, generally exhibit comparable analgesic effects across sexes when measured using conventional pain intensity scales (78). Nevertheless, women tend to report a higher incidence of adverse drug reactions, particularly with opioid medications (79). Women show greater sensitivity to side effects such as nausea, dizziness, constipation, and opioid-induced hyperalgesia. These differences may reflect sex-specific pharmacokinetics and pharmacodynamics, including variations in drug metabolism, receptor expression, and immune interactions (79).

### 5.2 Psychotherapeutic interventions

Evidence suggests that psychological therapies such as cognitive-behavioural therapy (CBT), acceptance and commitment therapy (ACT), and mindfulness-based approaches yield similar overall efficacy in men and women regarding pain reduction and functional improvement (80). While the therapeutic mechanisms may engage partly distinct emotional and cognitive circuits between sexes, current clinical outcome measures do not consistently capture these qualitative differences. Thus, psychotherapy remains effectively sex-agnostic in terms of measurable pain outcomes, even if underlying neural or affective processing diverges.

### 5.3 Surgical interventions

Surgical treatments for pain-related conditions do not show robust sex differences in long-term pain relief when evaluated with standard outcome assessments (53). However, complication profiles differ: men demonstrate a greater susceptibility to perioperative cardiovascular events and a higher likelihood of postoperative wound infections (81, 82). These disparities indicate that while surgical efficacy in terms of pain modulation may be similar across sexes, perioperative risk management requires sex-informed clinical strategies.

### 5.4 Neuromodulation

Neuromodulation techniques, including spinal cord stimulation (SCS) and deep brain stimulation for neuropathic pain, yield comparable analgesic outcomes between men and women when assessed using standard clinical pain reduction metrics (83). Recent large-scale matched cohort analyses confirm that the likelihood of receiving SCS therapy does not differ by sex in chronic pain due to persistent spinal pain syndrome (PSPS) when baseline clinical characteristics are balanced (84). However, sex-specific patterns emerge in device-related complications. Women demonstrate some higher rates of hardware-related revisions and explants, often linked to infection, discomfort, or perceived lack of therapeutic benefit (84). Notably, in the propensity-matched analysis of patients with PSPS, women exhibited a slightly but significantly higher risk of SCS explant despite receiving SCS at equal rates to men, and despite similar reported pain relief following implantation (84). This disconnect suggests that although neuromodulation achieves equivalent pain reduction across sexes, women may experience lower device tolerance or higher complication sensitivity, raising important considerations for sex-specific peri-implantation counselling, psychological screening, and postoperative support.

The apparent sex-agnostic nature of current treatments likely reflects the fact that existing therapies target core nociceptive and affective pathways that are shared across sexes, even if the upstream mechanisms that drive pain chronification differ (53). In other words, analgesic and neuromodulatory interventions converge on common neurobiological nodes—such as spinal dorsal horn processing, thalamocortical transmission, and descending modulatory circuits—that are present and functional in both men and women (13). Similarly, psychotherapeutic interventions act on shared cognitive-emotional regulatory networks, even if the emotional valence and stress responsivity differ between sexes.

While current treatments are designed to engage sex-indifferent mechanisms, the growing insight into sex-specific immune and neural pathways opens avenues for targeted adjunctive therapies. For example, male-biased microglial activation in neuropathic pain might be optimally addressed with microglia-specific modulators, whereas female-biased T-cell–driven mechanisms suggest potential benefit from immunotherapies that modulate adaptive immune responses (53). Combining these sex-specific strategies with existing sex-neutral treatments could enhance therapeutic precision, improve tolerability, and reduce complication rates. This integrated approach—pairing common pathway modulation with sex-specific adjuncts—represents a promising direction for future pain therapeutics (53).

## 6 Sociocultural contributions to sex-dependent differences in chronic pain

Biological explanations alone cannot fully account for the sex differences observed in pain and suffering. Social and cultural factors interact with biology in powerful ways to shape how pain is perceived, expressed, and treated (85). Gender norms influence not only the internal experience of pain but also how individuals communicate their suffering. Men are often encouraged to adopt stoicism, with cultural scripts that frame the open expression of pain as a sign of weakness (86). This can lead to underreporting of symptoms, delays in seeking medical attention, and potentially poorer outcomes. In contrast, women may encounter fewer social barriers to expressing pain, yet they often face another form of disadvantage: their pain is more likely to be dismissed as emotional or psychogenic by healthcare professionals (87). Such biases in clinical encounters contribute to diagnostic delays and under-treatment, particularly in conditions that disproportionately affect women, such as fibromyalgia, migraine, and endometriosis (87, 88). Moreover, cultural expectations surrounding women reproductive health can normalize suffering (85, 89). Labor pain, menstrual pain, pregnancy-related pain, and menopausal symptoms are frequently minimized or regarded as an unavoidable part of womanhood, reinforcing the idea that women should endure discomfort without complaint (90, 91). This normalization not only shapes women’s own perceptions of what constitutes “legitimate” pain but also influences how seriously their pain is taken in medical settings.

The intersection of sociocultural and biological factors creates a feedback loop in which women experience greater suffering (13, 25). Their heightened pain sensitivity and prevalence of chronic pain are compounded by societal expectations that either discourage medical attention or diminish the validity of their complaints when they do seek care. Consequently, sociocultural influences must be considered alongside neurobiological mechanisms to fully understand sex differences in pain.

## 7 Future directions for research

The recognition of sex differences in pain has gained momentum in recent years, but significant challenges remain. A priority for future work is the systematic integration of sex as a biological variable across all stages of research. Preclinical studies must consistently include both male and female animals, not only to identify sex-specific mechanisms but also to clarify shared pathways that transcend sex. Similarly, clinical trials should analyse outcomes by sex and, where possible, by hormonal stage, recognizing that puberty, pregnancy, menopause, and andropause each represent unique neuroendocrine contexts for pain processing.

Research must also move beyond biological mechanisms to incorporate the sociocultural environment in which pain is experienced. Gender roles, cultural expectations, and healthcare inequities shape both the reporting of pain and its treatment. Integrating qualitative methods with quantitative approaches may provide a richer understanding of how these cultural forces interact with biological vulnerability to influence the lived experience of pain.

Another critical direction involves bridging the gap between acute and chronic pain. Too often chronic pain is conceptualized as a simple extension of acute nociception, yet neuroplasticity, immune involvement, and psychosocial processes transform it into a distinct condition. Longitudinal studies following individuals from the onset of acute pain into chronic states could clarify how sex-specific factors contribute to chronification.

There is a pressing need to translate mechanistic insights into sex-specific therapies. The example of morphine metabolism demonstrates how neglecting sex can lead to inappropriate treatment strategies that disadvantage women (9). Developing tailored pharmacological, hormonal, and psychological interventions will require a more nuanced understanding of how genetic, immune, and hormonal mechanisms interact differently across sexes. By embracing this complexity, research can move toward precision medicine approaches that not only acknowledge sex differences but actively use them to optimize treatment outcomes.

The next step in therapeutic evolution may involve layered treatments: a sex-neutral core therapy (e.g., neuromodulation, SNRIs, CBT, opioid receptor agonists) targeting shared nociceptive circuits, combined with a sex-specific adjunct aimed at upstream modulators (53). Psychotherapy, though producing similar outcome metrics, may also operate through sex-specific circuitry: in women, limbic-prefrontal reappraisal alterations; in men, modulation of dorsal anterior cingulate and insula connectivity (13). Future stratified imaging-guided psychotherapy could tailor session content depending on sex-specific network engagement. This dual-framework model—common pathway engagement and sex-specific upstream modulation—aligns with precision medicine and leverages mechanistic insights into microglial vs. T-cell dominance, hormonal neuromodulatory differences, and immune–neural crosstalk documented in both neuropathic and nociplastic pain paradigms (53).

By explicitly integrating these insights into treatment algorithms, current sex-agnostic therapies can be refined rather than replaced, evolving into hybrid regimens that maintain universal efficacy while optimizing tolerability and complication profiles through sex-informed adjunctive strategies.

## 8 Conclusion

Women consistently demonstrate lower pain thresholds and tolerance, greater pain unpleasantness, and higher prevalence of chronic pain and comorbid affective disorders (24, 25). These differences arise from an interplay of genetic, hormonal, immunological, and neural mechanisms, particularly within the medial pain pathway and descending modulatory circuits. They also highlight the importance of considering sex as a fundamental biological variable in both basic and clinical research. Failure to account for sex differences not only obscures the true complexity of pain but risks perpetuating ineffective or harmful treatments. By integrating structural, functional, hormonal, and immunological perspectives, future work may develop sex-specific strategies for pain management—transforming a one-size-fits-all approach into personalized, biologically informed care. Future studies must move beyond prevalence and intensity differences to focus on qualitative mechanisms. By embracing sex as a biological variable risks and tailoring interventions to sex-divergent pathways, we can accelerate progress toward precision pain therapeutics.
